# Cytokine treatment in cancer immunotherapy

**DOI:** 10.18632/oncotarget.5095

**Published:** 2015-08-02

**Authors:** Michele Ardolino, Joy Hsu, David H. Raulet

**Affiliations:** Department of Molecular and Cell Biology, and Cancer Research Laboratory, Division of Immunology and Pathogenesis, University of California, Berkeley, Berkeley, CA, USA

**Keywords:** immunotherapy, cytokines, NK cells

In the interplay between cancer and immune system, tumor cells employ several mechanisms to evade the immune response [1]. One such mechanism is the suppression of MHC class I expression, which strongly reduces the antigenicity of tumor cells, therefore preventing an immune response mediated by cytotoxic T cells. Impaired MHC expression is generally common among tumors and in one systematic study was detected in the majority of breast cancer samples [3]. The immune system has evolved strategies to “sense” the lack of MHC class I expression on transformed cells. Indeed, MHC-deficient tumor cells induce a strong response mediated by Natural Killer (NK) cells, a cytotoxic subtype of innate lymphoid cells that play an important role in immunity against cancer [2]. Many tumors with impaired MHC class I expression are malignant and progress, however, suggesting that they have evaded control by the immune response [3]. In our recent publication in *The Journal of Clinical Investigation*, we found that NK cells become functionally anergic (i.e. show suppressed functional activity) after prolonged exposure to MHC-deficient tumor cells in vivo. Anergy was characterized by reduced ability to phosphorylate intracellular kinases, such as ERK1/2 or AKT, and to produce effector cytokines or to disgorge killer granules after ex vivo stimulation of the tumor infiltrating NK cells [4]. Therefore our results revealed a new mechanism employed by MHC-deficient tumors to escape from NK cell responses. These findings are in accord with previous studies showing that human NK cells are often dysfunctional in oncological patients [2].

In an effort to revert the functional anergy of NK cells within MHC-deficient tumors, we treated tumor-bearing mice with pro-inflammatory cytokines. Some mice received a combination of recombinant IL-12 and IL-18 whereas another cohort of mice was treated with a mutant form of IL-2 called a “superkine” [5], which binds with high affinity to the IL-2 receptor even when it lacks the receptor α-chain (CD25). Both treatments reverted the functional anergy of tumor infiltrating NK cells and significantly increased the survival of mice bearing MHC class I-deficient tumors [4]. In published studies, pro-inflammatory cytokines provided promising results in pre-clinical models and some partial efficacy in clinical trials of cancer patients, albeit with significant toxicity. In our hands, cytokine treatments were relatively non-toxic and increased the life-span of tumor-bearing animals. However, neither of the two treatments were sufficient to eradicate tumors in all animals once they were established, and, depending on the experiment, between 20 and 80% of the mice succumbed [4].

Recent advances in immuno-oncology have led to the development of drugs that boost T cell responses against tumors, based on the ability of T cells to recognize tumor neo-antigens [6]. However, MHC class I-deficient tumor cells cannot present neo-antigens and are poor targets for cytotoxic T cells, revealing the need for alternative strategies to marshal immune responses leading to tumor elimination. In light of our results, cytokine treatments should be re-explored in terms of efficacy vs toxicity, specifically in patients where the immune response against tumors is characterized by a strong NK cell component, and especially in cases where tumors exhibit MHC-deficiency.

Strategies to mobilize an NK cell response against tumors include induction of antibody-dependent cellular cytotoxicity or treatment with antibodies that target inhibitory receptors expressed on the surface of NK cells [7]. In a scenario where NK cell responses to tumor cells can be induced/boosted by such treatments, one potential caveat could be that tumor cells could still be able to induce functional anergy on NK cells, therefore reducing or abolishing the efficacy of the treatment. Cytokine therapy could be therefore combined with these treatments in order to prevent/revert the induction of NK cell anergy, and provide additional therapeutic benefit. The adverse effects caused by cytokine treatment could be circumvented by using engineered version of cytokines with reduced toxicity, such as the aforementioned IL-2 superkine. Furthermore, since cytokines would not be employed as a mono-agent, it is possible that lower, less toxic doses would suffice to prevent NK cell anergy.

Preclinical studies and a better understanding of the molecular pathways stimulated by immunotherapy will presumably provide additional support for employing cytokine therapy in combinatorial treatments for amplifying the NK-mediated anti-tumor response.

## References

[1] Mittal D (2014). Current Opinion in Immunology.

[2] Marcus A (2014). Adv. Immunol.

[3] Garrido F (2001). Adv. Cancer Res.

[4] Ardolino M (2014). J. Clin. Invest.

[5] Levin AM (2012). Nature.

[6] Schumacher TN (2015). Science.

[7] Vey N (2012). Blood.

